# Chitin Deacetylase Gene Family Positively Regulates the Accumulation of Rice Stripe Virus in *Laodelphax striatellus* Fallén (Hemiptera: Delphacidae) Ovaries

**DOI:** 10.3390/insects16040334

**Published:** 2025-03-22

**Authors:** Wenxing Hu, Ao You, Jiao Zhang, Yao Li, Shimin Zuo, Fang Liu, Lu Zhang

**Affiliations:** 1 College of Plant Protection, Yangzhou University, Yangzhou 225009, China; 2 Key Laboratory of Plant Functional Genomics of the Ministry of Education, Agricultural College, Yangzhou University, Yangzhou 225009, China; 3 Jiangsu Co-Innovation Center for Modern Production Technology of Grain Crops, Yangzhou University, Yangzhou 225009, China

**Keywords:** rice stripe virus, small brown planthopper, viral accumulation, vitellogenin receptor, reproduction

## Abstract

Chitin deacetylase modifies chitin and has critical functions in the growth and development of insects; however, whether it has other roles is unclear. *Laodelphax striatellus* not only directly feeds on the phloem sap of rice but also transmits RSV, leading to significant losses in rice production. In this study, four *CDA* genes were identified based on SBPH genomic data and were classified into groups I and III based on a phylogenetic analysis. The expression of *LsCDA1* and *LsCDA2* in RSV-infected SBPH was increased by 282% and 159%, respectively, relative to the non-infected SBPH control. Additionally, RSV enhanced the expression of *LsCDA1* (increased by 194%) in the ovaries. Yeast two-hybrid and glutathione-S-transferase pull-down assays demonstrated the interaction between LsCDA1 and RSV proteins NP and NS2. Furthermore, the knockdown of *LsCDA1* expression decreased viral loads in RSV-infected SBPH and its ovaries by 66% and 72% relative to the dsGFP control. Silencing *LsCDA1* significantly decreased *VgR* expression in SBPH and its ovaries and reduced fecundity. These results indicate that LsCDA1 positively regulates RSV accumulation in the ovaries and SBPH reproduction by modulating *VgR* expression, which offers a novel strategy for controlling both RSV and SBPH by targeting LsCDA1.

## Simple Summary

*Laodelphax striatellus* Fallén (Hemiptera: Delphacidae), the small brown planthopper (SBPH), transmits the rice stripe virus (RSV), causing substantial losses in rice production. In this study, we identified four Chitin deacetylase genes (*LsCDA1*~*LsCDA4*) from the genome of SBPH. RSV infection increased the expression of *LsCDA1* and *LsCDA2* in SBPH. Additionally, RSV enhanced the expression of *LsCDA1* in SBPH ovaries. The direct interaction between LsCDA1 and RSV proteins NP and NS2 was demonstrated. The inhibition of *LsCDA1* expression decreased viral accumulation in SBPH ovaries by regulating vitellogenin receptor (*VgR*) expression and completely suppressed SBPH reproduction. These results indicate that LsCDA1 positively regulates RSV accumulation in the ovaries and SBPH reproduction by altering *VgR* expression.

## 1. Introduction

The transmission of plant viruses by insects causes serious economic damage amounting to 30 billion dollars annually [1,2,3]. Insects transmit viruses via nonpersistent, semipersistent, circulative-nonpropagative and circulative-propagative routes [4,5]. Viruses that are transmitted via circulative-propagative means can invade insect ovaries for transovarial transmission [6], which is highly impactful on the epidemiology of plant viruses [7]. Few reports exist documenting the spread of arboviruses via transovarial transmission. In *Drosophila melanogaster*, the retrovirus ZAM relies on the vitellogenin receptor (VgR) for transmission into fly oocytes [8]. In the leafhopper *Nephotettix cincticeps*, the bacterial symbiont *Sulcia* mediates the entry of rice dwarf virus (RDV) into planthopper ovaries by exploiting the vitellogenin (Vg) protein [9]. In the interaction of the tomato yellow leaf curl virus (TYLCV) with the whitefly *Bemisia tabaci*, the viral coat protein interacts with Vg to facilitate entry into the insect ovaries [7]. Although the accumulation of arbovirus particles in the insect ovary requires the assistance of host Vg and VgR, it is not clear whether other host factors are exploited for transmission.

Chitin deacetylase modifies or degrades chitin-containing tissues by hydrolyzing chitin into chitosan, which impacts insect growth and development [10]. Interestingly, two studies on lepidopteran insects suggested that CDA regulates viral replication in the midgut. The *Spodoptera exigua* nuclear polyhedrosis virus (SeNPV) enhances the solubility and permeability of the *S. exigua* peritrophic membrane (PM) by promoting the expression of *CDA* to facilitate viral replication [11]. In contrast, *Heliothis armigera* lowers its susceptibility to baculovirus by reducing PM permeability by downregulating *HaCDA5a* [12]. Unfortunately, the role of CDAs in the accumulation of arboviruses by hemipteran insects is not well-studied and awaits further elucidation.

*Laodelphax striatellus*, commonly known as the small brown planthopper, is a vector for the rice stripe virus, which causes substantial losses in rice production [13]. RSV is a member of the genus *Tenuivirus* in the *Phenuiviridae* family [14]. The RSV genome is comprised of four RNAs: RNA1 encodes RNA-dependent RNA polymerase (RdRP); RNA2 encodes the RNA silencing suppressor NS2 and membrane glycoprotein NSvc2; RNA3 encodes the RNA silencing suppressor NS3 and nucleocapsid protein NP; and RNA4 encodes the disease-specific protein NS4 and movement protein NSvc4 [15,16,17,18]. SBPH transmits RSV in a circulative–propagative mode; the virus can even occur in SBPH ovaries and be transmitted from female adults to their progeny. Previous studies showed that the repression of *Vg* expression blocks RSV entry into SBPH ovaries [19,20] and demonstrated that RSV particles bind to Vg via a NP–Vg interaction in the SBPH hemolymph [20]. The RSV–Vg complex is ultimately assimilated into ovarian cells via VgR-mediated endocytosis [20,21]. Thus, VgR deficiency also inhibits RSV accumulation in the ovaries [21,22]. It is unclear whether the insect vector encodes other factors that mediate viral accumulation through Vg and VgR in SBPH ovaries.

In this report, the RSV–SBPH system was used to investigate the roles of insect CDAs in viral accumulation in the insect vector. Four *CDA* genes (*LsCDA1-LsCDA4*) were identified in the SBPH genome. RSV infection induced the expression of *LsCDA1* and *LsCDA2* in SBPH. Additionally, RSV enhanced the expression of *LsCDA1* in the ovaries of SBPH. An interaction between LsCDA1 and RSV proteins NP and NS2 was demonstrated through yeast two-hybrid (Y2H) and glutathione-s-transferase (GST) pull-down assays. LsCDA1 enhanced RSV accumulation in SBPH ovaries and promoted SBPH fecundity by regulating *VgR* expression. These results provide compelling evidence for the vital role of insect CDAs in the accumulation of arboviruses.

## 2. Materials and Methods

### 2.1. RSV, Plants and Insects

RSV-infected and non-infected SBPH were originally collected in Jiangsu, China, and propagated in our laboratory for 12 years. SBPHs were reared in an incubator at 27 ± 1 °C with a light/dark photoperiod of 16/8h and RH 70 ± 5%; insects were supplied with fresh rice seedlings every 10–12 d. To maintain a stable RSV infection rate in the RSV–SBPH population, single pairs of SBPH were isolated and placed in glass tubes until eggs were deposited. RSV infection in female SBPH from each pair was detected using Dot-ELISA with a monoclonal antibody against RSV NP (anti-NP, 1:500 dilution; Cat. No. MAB0007, Beijing Green Castle Agricultural Biotechnology Co., Ltd., Beijing, China) [6]. The offspring of RSV-infected female SBPH were subsequently used as the RSV–SBPH population.

### 2.2. Identification and Phylogenetic Analysis of CDAs

Genes encoding CDAs in SBPH were identified using InsectBase 2.0 (http://v2.insect-genome.com). The translational products of four *LsCDA* genes were identified with ORF finder (https://www.ncbi.nlm.nih.gov/orffinder/ (16 October 2024)) and queried with BLASTP (https://blast.ncbi.nlm.nih.gov). The Simple Modular Architectural Research Tool (SMART) (http://smart.embl-heidelberg.de/ (22 October 2024)) was used to detect domains in the deduced LsCDA proteins, and sequence alignments were generated with ClustalX [23]. Conserved domains were identified in CDAs with the NCBI Conserved Domain database (https://www.ncbi.nlm.nih.gov/Structure/cdd/wrpsb.cgi (25 October 2024)). Phylogenetic analyses of LsCDA and CDAs in other species were conducted with MEGA7 (https://www.megasoftware.net/) using the maximum likelihood algorithm and RAxML-NG (https://github.com/amkozlov/raxml-ng (12 November 2024)) with 1000 bootstrap replications.

### 2.3. RNA Extraction and Real-Time Quantitative PCR (RT-qPCR)

RNA was isolated from RSV-infected and non-infected SBPH female adults (n = 10), as well as dissected SBPH ovaries, midguts, salivary glands and hemolymph, using TRIzol reagent (Cat. No. R401-01; Vazyme, Nanjing, China). For tissue dissection, adult SBPHs (n = 100) were anesthetized using CO_2_ for 1–3 min and subsequently transferred to a 90 mm-diameter Petri dish. The forelegs were removed at the coxa-trochanter joint using forceps (WPI, Sarasota, FL, USA) to collect hemolymph. The remaining SBPH specimens were dissected to isolate the midgut, ovaries and salivary glands. The quality and concentration of extracted RNAs were calculated with a NanoDrop 2000c (Thermo Fisher Scientific, Waltham, MA, USA). cDNAs were synthesized from RNA samples following the removal of potential genomic DNA contamination using the HiScript II 1st Strand cDNA Synthesis Kit (Cat. No. R211-01; Vazyme, Nanjing, China). Primers were designed with Primer3 (https://primer3.ut.ee/ (15 October 2024)) and are provided in Supplementary Table S1.

Levels of *LsCDAs* (*LsCDA1*~*LsCDA4*), *Vg*, *VgR*, RSV *NP* and SBPH *actin* were measured using the primers shown in Supplementary Table S1. The primers for SBPH *actin* and RSV *NP* were consistent with previous literature [6]. The *LsCDAs*, *Vg* and *VgR* primers, based on their gene sequences, were shown to have an amplification efficiency close to 100% based on an analysis of the six-step 10-fold dilution sequence. RT-qPCR was conducted using a CFX96 Real-Time PCR System (Bio-Rad, Hercules, CA, USA) and IQ SYBR Green SuperMix (Cat. No. 1708880; Bio-Rad, Hercules, CA, USA) as follows: denaturation for 3 min at 95 °C, followed by 40 cycles at 95 °C for 10 s, and 60 °C for 30 s. Then, the RT-qPCR results were normalized to SBPH *actin*. Relative expression levels were determined using CFX Manager v.3.1 (Bio-Rad, Hercules, CA, USA) software. Experiments were executed with three replications, and mean values were determined as described previously [24].

### 2.4. Cloning and Characterization of LsCDA1

To obtain the *LsCDA1* full-length sequence, the cDNA of SBPH synthesized above was used as a template. The amplification was conducted with the specific primers listed in Supplementary Table S1. The amplified PCR product was cloned into a TA/Blunt-Zero vector (Cat. No. C601-02; Vazyme, Nanjing, China). The recombinant plasmid was then transformed into *Escherichia coli* (strain DH5α). The sequence of the *LsCDA1* fragment in the vector was confirmed via Sanger sequencing (Tsingke, Beijing, China) and deposited in NCBI (GenBank accession no. PQ898847). The successful constructed plasmid was consequently named T-LsCDA1. The predicted *LsCDA1* amino acid sequence was subjected to Blast analysis using DNAman 8.0 software (LynnonBiosoft, San Ramon, CA, USA) with the sequence collected from hemipteran and dipteran insects.

### 2.5. Yeast Two-Hybrid Assays

Potential interactions between LsCDA1 and RSV proteins (NS2, NSVc2, NS3, NP, SP and NSVc4) were investigated using Y2H assays. The sequences of each protein were amplified from the synthesized cDNA of RSV-infected SBPH with the specific primers (listed in Supplementary Table S1) containing adaptor sequences that overlap with vectors. The Easy Clone cDNA Library Construction Kit (Cat. No. P01010; Dualsystems Biotech AG, Shanghai, China) was used to clone those sequences into the linearized vectors (pGADT7 or pGBKT7) digested with *Nde1* and *BamH1* (Cat. no. R0111S and R0136S; NEB, Ipswich, MA, USA), which contain the GAL4 activation domain (AD) and GAL4 DNA-binding domain (BD), respectively. LsCDA1 cloned into pGADT7 were then designated as AD-LsCDA1, whereas RSV proteins cloned into pGBKT7 were designated as BD-RSV proteins (NS2, NSVc2, NS3, NP, SP and NSVc4). Yeast two-hybrid assays were then conducted following the protocols supplied with the Yeastmaker^TM^ Yeast Transformation System 2 (Cat. No. 630439, Takara-Bio, Shiga, Japan), and positive clones were selected on SD quadruple dropout (QDO) medium (SD/-Ade/-His/-Leu/-Trp) containing 4 mg mL^−1^ X-gal (5-bromo-4-chloro-3-indolyl-*α*-d-galactopyranoside).

### 2.6. GST Pull-Down Assays

*LsCDA1* cDNA fragments were amplified from T-LsCDA1 using specific primers (showed in Supplementary Table S1) with sequences that overlapped with pGEX-4T-1. The amplified product was subcloned into pGEX-4X-1 as glutathione-S-transferase (GST) translational fusions in the *N*-terminal of *LsCDA1* sequence to generate GST-LsCDA1. Similarly, RSV *NP* and *NS2* were cloned into pET-28a(+) with a 6X histidine tag to generate His-NP and His-NS2. Recombinant proteins were produced in *Escherichia coli* strain Rosetta and purified using His-Tag and GST Mag-Beads as recommended by the manufacturer (Cat. No. C650033 and C650031; Sangon Biotech, Shanghai, China). Equal amounts of purified proteins (GST-LsCDA1 with His-NP, and GST-LsCDA1 with His-NS2) were mixed and incubated in GST binding buffer (50 mM Tris, 150 mM NaCl, 0.1% Triton X-100, 1 mM PMSF, 1% protease inhibitor cocktail [pH 8.0]) for 16 h at 4 °C. As negative controls, His-NP or His-NS2 was separately incubated with GST protein alone. Beads were washed four times with pull-down buffer, and retained proteins were released by adding 2× loading buffer. The eluted proteins from the pull-down assay were then separated via SDS-PAGE and detected using Anti-GST (Cat. No. CSB-MA000031M1m; Cusabio, Wuhan, China) and Anti-His antisera (Cat. No. CSB-MA000159; Cusabio, Wuhan, China).

### 2.7. RNA Interference

The cDNA fragments of *LsCDA1* and green fluorescent protein (*GFP*; served as a control) containing T7 promoter sequences were amplified using the primers listed in Supplementary Table S1. The T7 RiboMAX™ Express RNAi System (Cat. No. P1700; Promega, Madison, WI, USA) was employed to synthesize double-stranded RNA (dsRNA) from the cDNA templates obtained above. The dsRNAs (36.8 ng) were injected into the haemocoel of SBPH using the Nanoliter 2010 Microinjection Pump (WPI, Sarasota, FL, USA). The gene silencing efficiencies were evaluated at 2 days post-injection (dpi) via RT-qPCR.

### 2.8. Western Blotting

The intact bodies of SBPH female adults and tissues, such as the ovaries, midguts, salivary glands and hemolymph were collected and lysed with TRIzol reagent for protein extraction. Proteins were then separated via 12% SDS-PAGE and transferred to PVDF membranes. Blots were probed with anti-GAPDH (1:2000 dilution; Cat. No. Ab157156; Abcam, Cambridge, UK) or anti-RSV NP (1:1000 dilution; provided by Dr. Kun Zhang, Yangzhou University, Yangzhou, Jiangsu Province, China). Immunoreactive bands were detected using a goat anti-rabbit/goat anti-mouse IgG-conjugated HRP antibody (Cat. No. CW0103S and CW0102S; CoWin Biosciences, Taizhou, China) at a 1:5000 dilution and imaged as reported previously [6]. Three samples were included for each experiment, and protein levels were calculated with Image Lab v. 5.2.1 (Bio-Rad).

### 2.9. Immunofluorescence Microscopy

Midguts, ovaries and salivary glands were dissected from dsLsCDA1- and dsGFP-treated SBPH females (n = 100). The dissected samples were fixed, blocked, incubated with preimmune serum and anti-RSV NP antisera at 1:500 and then incubated with Alexa Fluor 488-labeled secondary antibodies (Cat. No. 115-545-003; Jackson ImmunoResearch Laboratories, West Grove, PA, USA) as described [6]. Tissue samples were rinsed three times in PBS, stained with 100 nM DAPI and inspected for fluorescence using a Leica TCS SP8 STED confocal microscope (Leica, Wetzlar, Germany).

### 2.10. Evaluation of the Number of Offsprings and Egg Morphology

Emerging females injected with dsGFP or dsLsCDA1 were maintained on rice seedlings for 24 h, and healthy females (*n* = 45) were collected and individually placed in test tubes containing virgin males. At 15 days after pairing, offsprings were counted. Additionally, rice seedlings were randomly selected from each treatment group and dissected for egg collection at 2 and 5 dpi. Eggs from dsGFP- and dsLsCDA1-treated SBPH were then inspected and photographed.

### 2.11. Statistical Analysis

Statistical evaluations were executed with an unpaired *t*-test in GraphPad Prism 8 (version 8.0), and *p* values < 0.05 were considered significant. Datapoints represent the means ± SEM.

## 3. Results

### 3.1. Identification of CDA Genes in SBPH

**Figure 1:**
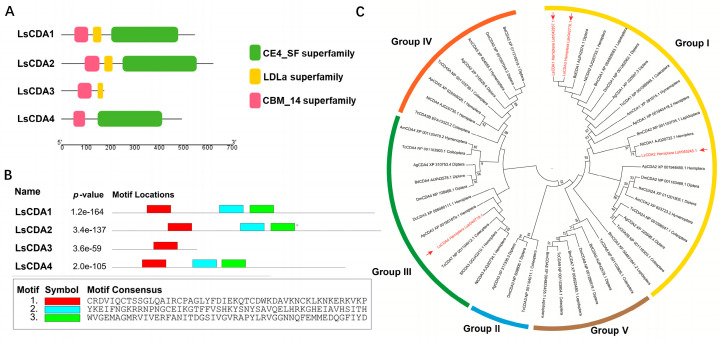
Domain architectures and phylogenetic analysis of LsCDAs. (**A**) Catalytic domains were identified in LsCDA proteins using the NCBI Conserved Domain search and are indicated by red, yellow and green rectangles, which represent the CBM_14, LDLa and CE4_SF superfamilies. (**B**) Location of conserved motifs within the CBM_14 (red) and LDLa (blue and green) domains of LsCDA1, LsCDA2, LsCDA3 and LsCDA4. Consensus sequences for the three motifs are shown below the diagram. (**C**) Phylogenetic analysis of *CDA* genes from *L. striatellus* and ten other insect species. The cluster was obtained based on the maximum likelihood algorithm using RAxML-NG with 1000 bootstrap replications. LsCDA proteins are labeled in red font. Abbreviations: Ls, *Laodelphax striatellus*; Am, *Apis mellifera*; Dm, *Drosophila melanogaster*; Nl, *Nilaparvata lugens*; Tc, *Tribolium castaneum*; Bd, *Bactrocena dorsalis*; Bm, *Bombyx mori*; Dc, *Diaphorina citri*; Ap, *Acyrthosiphon pisum*; Ag, *Anopheles gambiae*; Sf, *Sogatella furcifera*.

Four *CDA* genes were identified in the SBPH genome using InsectBase 2.0 and were designated *LsCDA1*, *LsCDA2*, *LsCDA3* and *LsCDA4*, which correspond to InsectBase accession numbers Lstr042837, Lstr045245, Lstr042776 and Lstr040719 (http://v2.insect-genome.com/). An analysis of the translational products revealed that all four deduced LsCDA proteins contained chitin-binding domains in the conserved CBM_14 superfamily. Furthermore, LsCDA1, LsCDA2 and LsCDA4 also contained a deacetylase catalytic domain in the CE4_SF superfamily, and LsCDA1, LsCDA2 and LsCDA3 possessed a low-density lipoprotein receptor domain in the LDLa superfamily (Figure 1A). Within the CBM_14 domains, a conserved motif (red rectangle, Figure 1B) was identified in all four CDA proteins. Two conserved motifs (blue and green rectangles, Figure 1B) were identified in the LDLa domains of LsCDA1, LsCDA2 and LsCDA4 (Figure 1B). These findings suggest that the catalytic functions of LsCDA proteins are highly conserved.

Insect CDAs were analyzed with MEGA7 and clustered into five phylogenetic groups (Group I−V; Figure 1C). SBPH proteins LsCDA1, LsCDA2 and LsCDA3 were present in Group I, and LsCDA4 was assigned to Group III along with ApCDA3, NlCDA3 and DcCDA3, which are hemipteran insects. These results further indicate that the structure and function of CDAs are highly conserved.

### 3.2. RSV Infection Induces LsCDA1 Expression in SBPH

**Figure 2:**
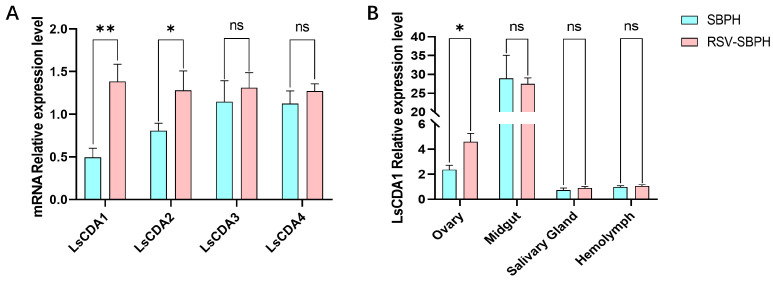
RSV infection alters *LsCDA1* expression. (**A**) RT-qPCR analyzed the expression levels of *LsCDA1, LsCDA2, LsCDA3* and *LsCDA4* in the intact body of RSV-infected SBPH (RSV-SBPH) and non-infected SBPH (SBPH), with four biological replicates for each group (n = 10). (**B**) RT-qPCR analyzed the expression levels of *LsCDA1* expression in the ovaries, midguts, salivary glands and hemolymph of non-infected and RSV-infected SBPH (RSV-SBPH), with three biological replicates for each group (n = 100). **, *p* < 0.01; *, *p* < 0.05; ns, nonsignificant.

To investigate whether LsCDAs function in the RSV infection of SBPH, the expression levels of *LsCDAs* were compared in non-infected and RSV-infected SBPH. RT-qPCR analysis revealed that the expression levels of *LsCDA1* and *LsCDA2* were significantly upregulated by 282% and 159%, respectively, in RSV-infected SBPH compared to the non-infected control (Figure 2A). No significant differences were observed for *LsCDA3* and *LsCDA4* expression in RSV-infected SBPH and the non-infected control (Figure 2A). Considering that *LsCDA1* exhibited more significant upregulation than *LsCDA2*, we initially focused on analyzing the role of LsCDA1 in RSV accumulation. Thus, we measured *LsCDA1* expression in the ovaries, midguts, salivary glands and hemocytes of RSV-infected and non-infected SBPH. *LsCDA1* expression was significantly upregulated in the ovaries of RSV-infected SBPH and was 194% higher than expression in non-infected SBPH (*LsCDA1*). No significant differences in expression were observed for *LsCDA1* in the midguts, salivary glands and hemocytes.

Next, we amplified the full-length *LsCDA1*sequence. Sequencing results showed that the full-length *LsCDA1* is 1653 bp in length and is predicted to encode a 551 kDa protein. The deduced *LsCDA1* amino acid sequence was compared with *CDAs* from other hemipteran pests, including *Nilaparvata lugens* and *Sogatella furcifera*, and the dipteran insects *Anopheles albimanus* and *Bradysia coprophila*. Multiple sequence alignments indicated that the hemipteran *CDAs* shared 96.23% and 96.91% amino acid identity with *LsCDA1*, respectively. Dipteran *CDAs* shared 87.66% and 88.02% amino acid identity with *LsCDA1*, respectively (Supplementary Figure S1).

### 3.3. RSV NP and NS2 Interact with LsCDA1 In Vitro

**Figure 3:**
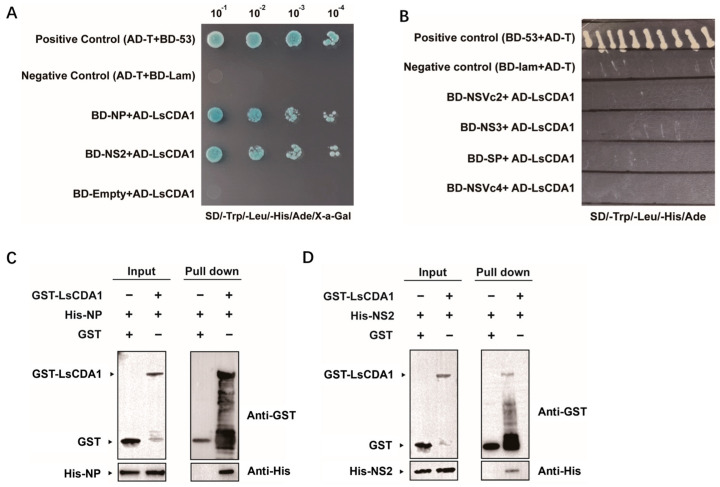
SBPH LsCDA1 interacts with RSV NP and NS2. (**A**) Interactions between AD-LsCDA1 and RSV BD-NP and BD-NS2 (bait domain, BD) in Y2H assays. Yeast cells containing the cloned genes were plated on quadruple dropout (SD/-Ade/-His/-Leu/-Trp) medium, and colonies were analyzed for β-galactosidase activity. Positive control, BD-53+AD-T; negative control, BD-lam+AD-T. The activation (AD) and bait (BD) domains were present in pGADT7 and pGBKT7, respectively. (**B**) Interaction screening between AD-LsCDA1 and RSV proteins (NSVc2, NS3, SP and NSVc4) in Y2H assays. Pull-down assays with GST-LsCDA1 and His-tagged NP (**C**) and His-tagged NS2 (**D**). Proteins were overproduced in *E. coli* and purified using His-Tag and GST Mag-Beads. GST-LsCDA1 was incubated with His-NP or His-NS2. Proteins were released from beads, separated via SDS-PAGE, and immunoblotted with anti-GST and anti-His antibodies.

Yeast two-hybrid assays were conducted to evaluate whether RSV proteins interact with LsCDA1. Yeast cells were co-transformed with the AD-LsCDA1 fusion and BD-RSV proteins fusion, and positive co-transformations were subsequently selected on SD/-Trp/-Leu/-His/-Ade/X-a-Gal medium. In this assay, the AD-LsCDA1 fusion did not interact with the BD-empty vector control. The results demonstrated that both RSV NP and NS2 interact with LsCDA1 (Figure 3A). Conversely, NSVC2, NS3, SP and NSVC4 did not interact with LsCDA1 (Figure 3B).

Further validation of the interaction between LsCDA1 and RSV NP or NS2 was investigated in a pull-down assay using GST-tagged LsCDA1 (GST-LsCDA1) and His-tagged NP and NS2. Purified GST-LsCDA1 bound to both His-tagged NP (Figure 3C) and NS2 (Figure 3D), thus confirming the results obtained in Y2H assays.

### 3.4. LsCDA1 Facilitates RSV Accumulation in Ovaries and Promotes SBPH Fecundity by Inducing VgR Expression

**Figure 4:**
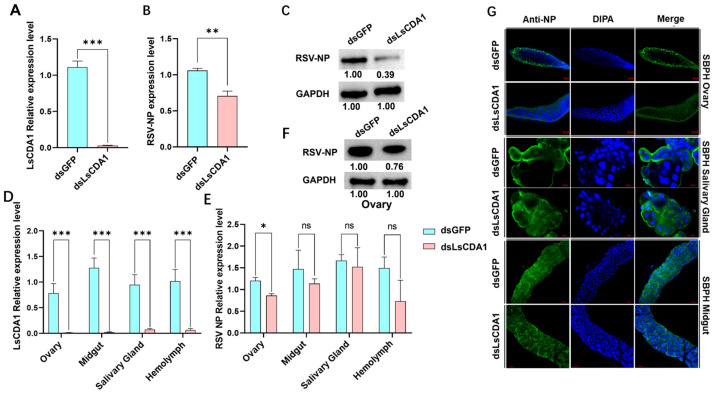
LsCDA1 facilitates RSV accumulation in the ovaries of SBPH. Expression of (**A**) *LsCDA1* and (**B**) RSV *NP* in dsGFP- and dsLsCDA1-treated SBPH infected with RSV. Each treatment was conducted with four biological replicates (n = 10). (**C**) Immunoblot analysis of NP and GAPDH levels in dsGFP- and dsLsCDA1-treated SBPH female adults infected with RSV. Blots were probed with anti-NP or anti-GAPDH antibodies. Relative expression of (**D**) *LsCDA1* and (**E**) RSV *NP* in the ovaries, midguts, salivary glands and hemolymph of RSV-infected SBPH. Each treatment was conducted with three biological replicates (n = 100). (**F**) Immunoblot analysis of NP and GAPDH levels in the ovaries of SBPH adult females treated with dsLsCDA1or dsGFP. Immunoblots were probed with anti-GAPDH or anti-NP antibodies. (**G**) Immunofluorescence assays of SBPH ovaries, salivary glands and midguts using anti-NP antisera. SBPH were treated with dsLsCDA1 or dsGFP. RSV NP was labeled with antisera conjugated to Alexa Fluor 488 (green). Nuclei were stained with DAPI (blue). Scale bar, 20 μm. *, *p* < 0.05; **, *p* < 0.01; ***, *p* < 0.001; ns, nonsignificant.

RNAi experiments indicated that *LsCDA1* expression in RSV-infected SBPH was suppressed at 48 h after dsLsCDA1 injection (Figure 4A). To evaluate RSV titers after dsLsCDA1 treatment, the expression of *NP* was evaluated via RT-qPCR. RSV loads in SBPH injected with dsLsCDA1 were significantly reduced by 66% compared with the dsGFP control (Figure 4B), which was further confirmed through immunoblotting using anti-NP antisera (Figure 4C).

To evaluate the tissues involved in the LsCDA1-mediated regulation of RSV titers, dsLsCDA1- and dsGFP-treated SBPH were dissected, and samples of ovaries, midguts, salivary glands and hemolymph were used to analyze *LsCDA1* and RSV *NP* expression. RT-qPCR confirmed that the suppression of *LsCDA1* mediated by RNAi reduced *LsCDA1* expression levels in all tissues (Figure 4D). However, when the *NP* expression was monitored in dsLsCDA1-treated tissue samples, NP transcripts were significantly reduced by 72% in ovaries but not in the other tissues (Figure 4E). Immunoblots confirmed that NP protein levels in the ovaries of SBPH adult females treated with dsLsCDA1 were substantially lower than levels in dsGFP-treated ovaries (Figure 4F), and this finding was confirmed in the immunofluorescence assays (Figure 4G). No significant changes were detected in other tissues. These results indicate that LsCDA1 facilitates RSV accumulation in SBPH and its ovaries.

**Figure 5:**
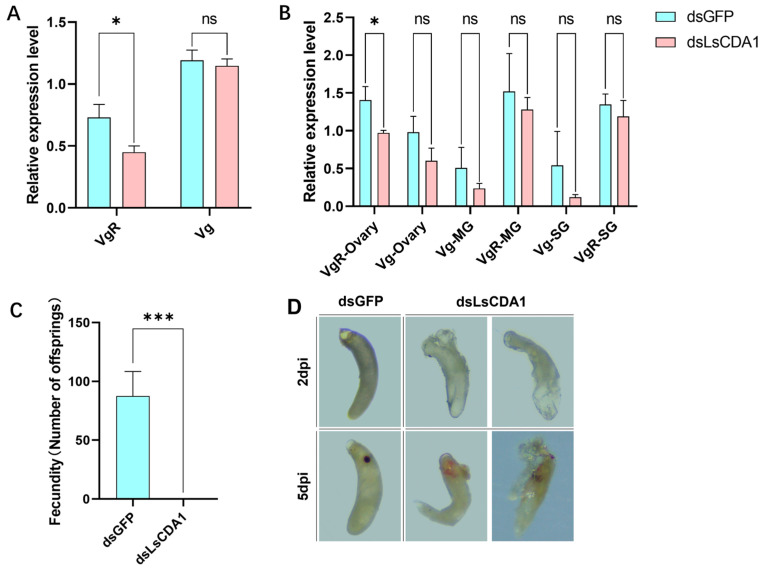
LsCDA1 promotes *VgR* expression and facilitates SBPH reproduction. (**A**) RT-qPCR analysis of vitellogenin (*Vg*) and vitellogenin receptor (*VgR*) expression in dsGFP- and dsLsCDA1-treated SBPH infected with RSV. Each treatment was conducted with three biological replicates (n = 10). (**B**) Relative expression of *Vg* and *VgR* in ovaries, midguts (MG) and salivary glands (SG) of RSV-infected SBPH treated with dsGFP and dsLsCDA1. Each treatment was conducted with three biological replicates (n = 100). (**C**) Numbers of progeny collected from female SBPH treated with dsGFP and dsLsCDA1. Each treatment was repeat forty-five times. (**D**) Egg morphology in SBPH females treated with dsGFP and dsLsCDA1. *, *p* < 0.05; ***, *p* < 0.001; ns, nonsignificant; days post-injection (dpi).

Considering the critical role of Vg and VgR in the entry of RSV into SBPH ovaries, we speculated that these genes may function in LsCDA1-mediated RSV accumulation in SPBH ovaries. To test this hypothesis, we examined *Vg* and *VgR* expression levels in dsGFP- and dsLsCDA1-treated SBPH. The expression of *VgR* was downregulated by 62% in intact females (Figure 5A) and 67% in ovaries (Figure 5B). Since VgR has a significant role in the transovarial transmission of RSV, we next assessed the vertical transmission rate and fecundity of RSV-infected SBPH following *LsCDA1* silencing. Offspring were not produced by dsLsCDA1-treated female adults but were collected at normal levels from dsGFP-treated females (Figure 5C). Eggs had a normal morphology in dsGFP-treated SBPH but displayed embryonic dysplasia in dsLsCDA1-treated SBPH (Figure 5D). These observations suggest that LsCDA1 facilitates RSV accumulation in the ovary through the Vg–VgR pathway and contributes to SBPH reproduction.

## 4. Discussion

*CDA* genes from arthropods were classified into orthologous Groups I–V based on phylogenetic relationships and conserved motifs [25,26]. Only Group I CDA from the black tiger shrimp *Penaeus monodon* and Group V CDA from lepidopteran insects were previously reported to function in virus accumulation. In *P. monodon*, CDA1 (phylogenetic Group I) exhibited putative immune functions against White spot syndrome virus (WSSV) [27]. In lepidopteran insects, midgut-specific CDA (phylogenetic Group V) was reported to have roles in viral replication [11,12]. In the current study, LsCDA1, LsCDA2 and LsCDA3 were assigned to Group I, and LsCDA4 was classified into Group III (Figure 1C). In response to RSV infection, *LsCDA1* and *LsCDA2* showed a significant increase in expression in SBPH. Moreover, *LsCDA2* showed a significant upregulation in its ovaries following RSV infection (Figure 2A,B). The RNAi-mediated silencing of *LsCDA1* decreased RSV accumulation in SBPH and its ovaries (Figure 4). These results implied that RSV infection induced *LsCDA1* expression and that elevated levels of *LsCDA1* facilitate RSV accumulation in SBPH ovaries. These assumptions were further supported by the results of Y2H and GST-pulldown assays, which demonstrated that RSV NP and NS2 interact with LsCDA1 (Figure 3). Thus, our data provided convincing evidence that insect CDA (phylogenetic Group I) is directly involved in arbovirus accumulation.

Prior studies revealed that Vg and VgR are essential for RSV entry and accumulation in SBPH ovaries [19,20,21,22,28]. In this study, we demonstrated that LsCDA1 promotes *VgR* expression in SBPH and its ovaries (Figure 5A,B), implying that increased RSV loads mediated by LsCDA1 may be associated with *VgR* expression. One study reported that CDA promotes fatty acid metabolism in *Diaphorina citri* [29], and another showed that fatty acid synthetase regulates the expression of *Vg* and *VgR* in SBPH [30]. Hence, we speculated that LsCDA1 promotes RSV accumulation in the ovaries of SBPH by modulating *VgR* expression via fatty acid metabolism. However, our speculation warrants further investigation.

Vg and VgR are essential in insect embryonic development and reproduction [31]. Our results showed that the fecundity of SBPH was inhibited by the embryonic dysplasia in dsLsCDA1-treated female parents (Figure 5C,D), suggesting that LsCDA1 is essential for SBPH reproduction by regulating *VgR* expression. In *D. melanogaster*, *serpentine* (*Serp*) and *vermiform (Verm)*, which are Group I homologs of *LsCDA1,* control tracheal tube development [29]. Knocking out of *Serp* and *Verm* resulted in embryonic lethal [32]. Accordingly, our results suggest that CDA likely functions in embryonic development and highlights the importance of LsCDA1 in VgR-mediated SBPH reproduction.

In summary, our findings demonstrate the direct interaction between LsCDA1 and RSV proteins NP and NS2. RSV infection induced *LsCDA1* transcription in SBPH, and the increased levels of LsCDA1 promote RSV accumulation in the ovaries by regulating *VgR* expression. In this context, LsCDA1 functions as a novel host factor that induces *VgR* expression to promote viral accumulation in the ovaries. Our study provides a new paradigm for understanding insect vector–plant virus interactions and offers a possible new target for the control of both SBPH and RSV.

## Data Availability

The original contributions presented in this study are included in the article/Supplementary Material. Further inquiries can be directed to the corresponding authors.

## Supplementary Materials

The following supporting information can be downloaded at: https://www.mdpi.com/article/10.3390/insects16040334/s1, Supplementary Table S1: Primers used in this study; Supplementary Figure S1: Multiple sequence alignment of *LsCDA1* and *CDAs* from hemipteran and dipteran insects. Abbreviations: *LCDA1*, PQ898847 from *Laodelphax striatellus*; *NlCDA2*, AJQ20733.1 from *Nilaparvata lugens*; *SfCDA2*, QQJ42209.1 from *Sogatella furcifera*; *AbCDA1*, XP_058063118.1 from *Anopheles bellator*; and *BcCDA1*, XP_037043829.1 from *Bradysia coprophila*. Amino acid residues shaded in black are identical among the five *CDA* sequences; those in gray are similar.
