# Supplementary material for: Chitin Deacetylase Gene Family Positively Regulates the Accumulation of Rice Stripe Virus in Laodelphax striatellus Fallén (Hemiptera: Delphacidae) Ovaries
Source: Insects. 2025 Mar 22;16(4):334. doi: 10.3390/insects16040334 (PMC12027493; doi:10.3390/insects16040334)
Supplement: Supplementary file 1 [file insects-16-00334-s001.zip › insects-3485978-supplementary.pdf]

insects

MDPI

Supplementary Materials

Chitin deacetylase gene family positively regulates the accumulation of rice stripe virus in *Laodelphax striatellus* Fallén (Hemiptera: Delphacidae) ovaries

Wenxing Hu[1], Ao You[1], Jiao Zhang[1], Yao Li[1], Shimin Zuo[2], Fang Liu[1,3*], Lu Zhang[1*]

Supplementary Table S1.

Primer Name	Sequence	Note
Vg-qPCR-F	CTGCTGGTTGGAGTCGGATT	RT-qPCR for Vg
Vg-qPCR-R	AATCCGACTCCAACCAGCAG	RT-qPCR for Vg
VgR-qPCR-F	GGCCGATTCTGTCACCAAGA	RT-qPCR for VgR
VgR-qPCR-R	GTCGGTTGGTACGTCAGGAG	RT-qPCR for VgR
q-RSV-F	TGAAAGTGGCGGCTGGAA	RT-qPCR for RSV-NP
q-RSV-R	CCACCGAGGACACTATCCCATA	RT-qPCR for RSV-NP
LsCDA1-qPCR-F	AAGTACGCCAACCTGACAGA	RT-qPCR for LsCDA1
LsCDA1-qPCR-R	CGTGATGGTGGAGTCGTAGA	RT-qPCR for LsCDA1
LsCDA2-qPCR-F	CCCTACATGATGCCCTTCCA	RT-qPCR for LsCDA2
LsCDA2-qPCR-R	GCTCCGTCATCCATGTCAAC	RT-qPCR for LsCDA2
LsCDA3-qPCR-F	GAAGTGCGATGTCAAGGGTC	RT-qPCR for LsCDA3
LsCDA3-qPCR-R	ATCCAGGGGTAGTTGTTGGG	RT-qPCR for LsCDA3
LsCDA4-qPCR-F	GACGTCATCCAGTGCACATC	RT-qPCR for LsCDA4
LsCDA4-qPCR-R	TGCATCTTTCCAGTCGCAAG	RT-qPCR for LsCDA4
Ls-Actin-F	GTCTCACACACAGTCCCCATCTATG	RT-qPCR
Ls-Actin-R	TCGGTCAAGTCACGACCAGC	RT-qPCR
LsCDA1-Full-F	ATGGCGTGGATGCGAACG	Full length amplification
LsCDA1-Full-R	TTAATTCGCCCTTAATCCGTCTCCGGTGGGG	Full length amplification
LsCDA1-T7-Ri-F	TAATACGACTCACTATAGGTA CTTCCGGATGCCTCACAG	dsRNA synthesis
LsCDA1-T7-Ri-R	TAATACGACTCACTATAGGCAC CTCGTCGATCCAGTACA	dsRNA synthesis
GFP-T7-Ri-F	TAATACGACTCACTATAGGGAAGGGCGAGGAGCTGTTCACCG	dsRNA synthesis
GFP-T7-Ri-R	TAATACGACTCACTATAGGGCAGCAGGACCATGTGATCGCGC	dsRNA synthesis
LsCDA1-AD-F	GACGTACCAGATTACGCTCAT ATGGCGTGGATGCGAACG	AD-LsCDA1 construction
LsCDA1-AD-R	GCAGCTCGAGCTCGATGGATCCATTCGCCCTTAATCCGTCTCCGGTGGGG	AD-LsCDA1 construction
BD-NS2-F	ATCTCAGAGGAGGACCTGCATATGGCATTACTCCTCTTCAAT	BD-NS2 construction

BD-NS2-R	GCCGCTGCAGGTCGACGGATCCTCACATTAGAATAGGGCACTCAT	BD-NS2 construction
BD-NSVc2-F	ATCTCAGAGGAGGACCTGCATATGCATTTTAAATCATATTTCATCT	BD-NSVc2 construction
BD-NSVc2-R	GCCGCTGCAGGTCGACGGATCCTTAATCAACCTGTCTGATGT CATT	BD-NSVc2 construction
BD-NS3-F	ATCTCAGAGGAGGACCTGCATATGAACGTGTTCACATCGTCT	BD-NS3 construction
BD-NS3-R	GCCGCTGCAGGTCGACGGATCCCTACAGCACAGCTGGAGAGC	BD-NS3 construction
BD-NP-F	ATCTCAGAGGAGGACCTGCATATGGGCACCAACAAGCCA	BD-NP construction
BD- NP-R	GCCGCTGCAGGTCGACGGATCCCTAGTCATCTGCACCTTCTG C	BD-NP construction
BD-SP-F	ATCTCAGAGGAGGACCTGCATATGCAAGACGTACAAAGGAC	BD-SP construction
BD-SP-R	GCCGCTGCAGGTCGACGGATCCCTATGTTTTGTGTAGAAGAG GTTG	BD-SP construction
BD-NSVc4-F	ATCTCAGAGGAGGACCTGCATATGCATTTTAAATCATATTTCATCT	BD- NSVc4 construction
BD- NSVc4-R	GCCGCTGCAGGTCGACGGATCCTTAATCAACCTGTCTGATGT CATT	BD- NSVc4 construction
LsCDA1-pGEX-F	GATCTGGTTCCGCGTGGATCCATGGCGTGGATGCGAACG	GST- LsCDA1 construction
LsCDA1-pGEX-R	TCAGTCAGTCACGATGCGGCCGCAAT CCGTCTCCGGTGGGG	GST- LsCDA1 construction
NS2-pET-F	CTGGTGCCGCGCGGCAGCCAT ATGGCATTACTCCTCTTCAATG	His- NS2 construction
NS2-pET-R	ACGGAGCTCGAATTCGGATCC TCACATTAGAATAGGGCACTCAT	His- NS2 construction
NP-pET-F	CTGGTGCCGCGCGGCAGCCAT ATGGGCACCAACAAGCCAG	His- NP construction
NP-pET-R	ACGGAGCTCGAATTCGGATCC CTAGTCATCTGCACCTTCTGCCT	His- NP construction

Supplementary Figure S1.

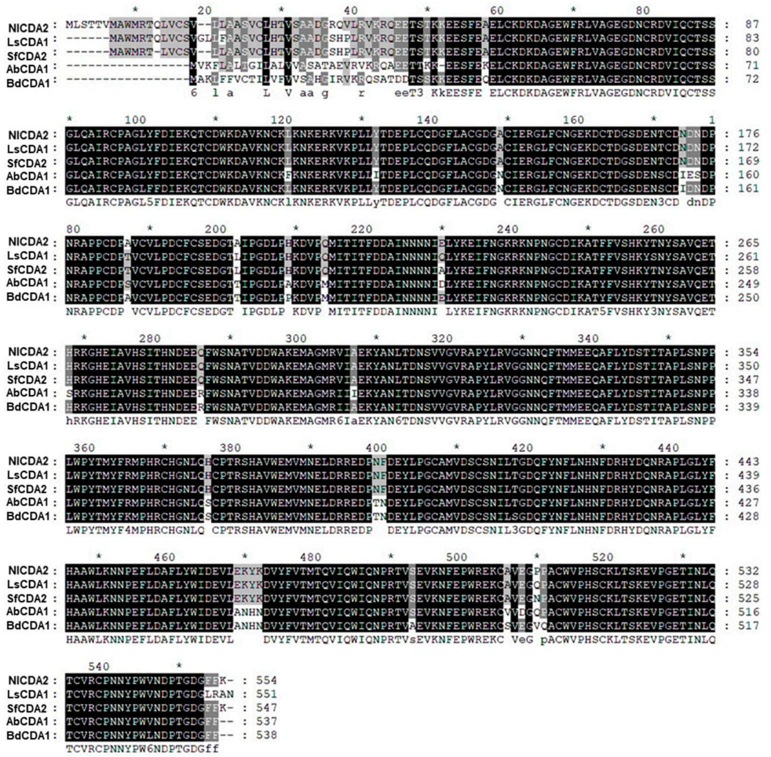

Supplementary Figure S1. Multiple sequence alignment of *LsCDA1* and *CDAs* from hemipteran and dipteran insects. Abbreviations: *LCDA1*, PQ898847 from *Laodelphax striatellus*; *NlCDA2*, AJQ20733.1 from *Nilaparvata lugens*; *SfCDA2*, QQJ42209.1 from *Sogatella furcifera*; *AbCDA1*, XP_058063118.1 from *Anopheles bellator*; and *BcCDA1*, XP_037043829.1 from *Bradysia coprophila*. Amino acid residues shaded in black are identical among the five *CDA* sequences; those in gray are similar.
